# Influence of the preference for sons on contraceptive use in Bangladesh: A multivariate analysis

**DOI:** 10.1016/j.heliyon.2020.e05120

**Published:** 2020-10-07

**Authors:** Mohammad Nazmul Hoq

**Affiliations:** Department of Business Administration, International Islamic University Chittagong, Bangladesh

**Keywords:** Contraception, Preference for sons, Socioeconomic and demographic variables, Bangladesh, Statistics, Public health, Reproductive system, Education, Health education

## Abstract

The preference for sons has been documented not only in Bangladesh but also in many Asian and African countries across various religions and social strata. This paper examines the levels, trends, and differentials in the use of contraceptives and investigates the effects of the preference for sons on contraception in Bangladesh. This research project extracted data from the last four nationally representative Bangladesh Demographic and Health Survey datasets (BDHS: 2004, 2007, 2011, and 2014) to examine the trends of preference for sons. The use of contraceptives among the respondents who had only daughters was comparatively lower than among those who had only sons due to a preference for sons. The analysis also reveals that the preference for sons was invariant with regards to various demographic and socioeconomic factors. Moreover, the analysis of religion in all four survey datasets indicates that Muslim women had stronger preferences for sons than their non-Muslim counterparts. Furthermore, the analysis showed that the preference for sons was strongest among women who had no education, did not work, and lived in rural areas.

## 1 Introduction

Bangladesh is one of the most densely populated countries in the world, with a growth rate of 1.37 per annum (BBS, 2015). Sustainable development during the last two decades has improved Bangladesh's economy, but the population growth rate has frustrated all development efforts. The government of Bangladesh has declared that population growth is the number one problem for the country; however, attempts to control this rapid growth rate have not been very successful. The findings of many research studies indicate that the main reason behind the high population growth rate is the low use of contraceptives, especially in the rural areas where the vast majority of the country's population lives (Mahmood and Ringheim, 1996; Dasgumpta et al., 2015). This low Contraceptive Prevalence Rate (CPR) can be attributed to the preference for sons and many different social, economic, cultural, and demographic factors. Moreover, the use of contraception is affected by the gender composition and number of living children. Furthermore, the reproductive behavior of parents is more or less affected by gender preferences in children.

The preference for sons is somewhat more pervasive among rural women, especially among practitioners of the birth spacing technique. Some socioeconomic factors, such as the training of the spouse and occupation, have been found to have a negative impact on fertility and a positive effect on contraceptive use among rural women (Appunni, 2006). It has also been found that the preference for sons is the primary consideration when permanent contraception is being considered in every southeast Asian nation. The affiliation is exceptionally solid in Nepal, India, and Pakistan. Women with fewer than two children are, by and large, considerably less to avail themselves of permanent contraception. On the other hand, the preference for sons has no real relationship with the choice in traditional and temporary contraceptive methods in this region (Channon, 2015). In addition, women in rural areas with high infant mortality rates are less likely to use contraceptives than women in urban areas with low infant mortality rates (Thang et al., 1992).

The preference for sons is not a new issue in most societies, and sons often receive preferential treatment in developing countries, especially South Asia (Bairagi and Langsten, 1986). The factors influencing a preference for sons include incomes earned as heads of families; fewer opportunities for females to obtain jobs and generate income outside the home; poor literacy rates, especially among women; providing security for parents in old age; remaining in the family; cultural restrictions on women; and cultural beliefs. Moreover, discrimination against girls increases their childhood mortality rate, which, in turn, alters the gender composition of the surviving children (Costa et al., 2017). Family units’ weighs couples who have son and another continue to put invest into women to keep having children until they produce a son. Previous findings have demonstrated that the notion that a son will provide for his parents in their old age is undoubtedly connected to the idea that having a son is imperative (Leung and Zhang, 2008; Begum et al., 2018).

The preference for sons has been an essential issue in demographic studies on developing nations for quite a while. In societies with high fertility regimes, gender preference can vary from region to region. This variation in gender preference can be due to religion, social status, or ethnic group. In developing countries, young mothers are reluctant to state that they must have a son, uncovering an inconsistency between the conceptive beliefs they express and their subsequent conduct (Brunson, 2010). Parents frequently feel that a daughter will usually move in with her in-laws after marriage; hence, they view explicit interest in a girl as being ineffective. In their view, teaching girls is akin to planting seeds in a neighbor's garden. Some parents think that daughters are destined to be ‘other people's property.’ Sons, on the other hand, are considered to be resources deserving of both short- and long-term investments (Sekher et al., 2010). Sex and social standards regularly constrain the reproductive decisions young women make to such an extent that they are forced to control their procreative conduct in such a manner as to reach a certain family size or composition of boys and girls to please influential elders in the family (Barua and Kurz, 2001; Barua et al., 2004). Hence, women may be "permitted" to undergo sterilization once they have produced the necessary number of sons. In a few nations with stable sex preferences, couples stop producing children only after they are happy with the composition of boys and girls, or, commonly, after the birth of a son. McClelland (1979) hypothesized that while couples may prefer a higher number of sons, those who already have more daughters may terminate childbearing earlier over concerns that the next child, if female, will worsen the existing ratio of boys and girls. The crux of this hypothesis, known as the risk hypothesis, is that an additional daughter's perceived disadvantage outweighs another son's perceived benefit.

In this investigation, an effort has been made to inspect the impact of the preference for sons on use of contraceptives in Bangladesh. Numerous factors influence the use of contraception. However, more attention must be paid to discovering how the preference for sons changes contraceptive use in conjunction with various socioeconomic and demographic factors. This study examined the possible factors responsible for the preference for sons and its effect on the use of contraceptives use because contraception is one of the proximate determinants of fertility, which determines population growth.

## 2 Materials and methods

This examination utilized information from four Bangladesh Demographic and Health Survey datasets (BDHS-2004, 2007, 2011, and 2014). These datasets were gathered for the Government of Bangladesh by the National Institute for Population Research and Training (NIPORT), with financing from the United States Agency for International Development (USAID)/Dhaka (NIPORT, 2005, NIPORT, 2009, NIPORT, 2012, NIPORT, 2016).

The dependent variable in the current examination is the current use of contraception. Hence, any female respondent (or her life partner) using a family planning method at the time of the study was viewed as a current user. The study's independent variables were the respondent's level of education, employment status of the respondent, husband's education, religion, socioeconomic status, access to mass media, place of residence, and number of living children.

The principal aim of the study was to measure the preference for sons. In the analysis, the proportion of surviving daughters was compared with the proportion of surviving sons. The number of surviving children included both children living at home and away from home at the time of the survey. Multivariate logistic regression analysis was used to identify how different categorical socioeconomic and demographic variables affected the preference for sons over daughters as well as the use of contraception. Consequently, logistic regression analysis was used to examine the association of (categorical or continuous) independent variable(s) with the dichotomous dependent variable.

## 3 Trends in the current use of contraceptive methods

This study's main objective was to observe the respondents' current use of contraception in different demographical health survey year, but the analysis was extended further back with the help of the Contraceptive Prevalence Survey (CPS), and the Bangladesh Fertility Survey (BFS) (NIPORT, 2001; Mitra et al., 1993; MOHPC, 1978). Fertility in Bangladesh has declines to a great extent due to the accessibility to modern contraceptives. In Bangladesh, contraception among married women had increased gradually from 8 percent in 1975 to 62.4 percent in 2014, an almost eightfold increase in less than four decades (Table 1). The use of contraceptives increased by 6.6 percent between 2007 and 2014 alone. The most frequently used contraceptive was the pill. In 1975, 2.7 percent of women were using the pill. This figure jumped to 28.5 percent in 2007 but declined to 27.0 percent in 2014. The next most frequently used class of contraceptives were the injectables, for which use increased from 0.5 percent in 1985 to 12.4 percent in 2014, with a dip in 2007. The next most frequently used contraceptive methods were condoms and female sterilization.

**Table 1:** Percentages of currently married women aged 10–49 using specific family planning methods, Bangladesh 1975–2014.

Method	1975 BFS	1985 CPS	1991 CPS	1996-97 BDHS	1999–2000 BDHS	2004 BDHS	2007 BDHS	2011 BDHS	2014 BDHS
Any Method	7.7	25.3	39.9	49.2	53.8	58.1	55.8	61.2	62.4
Any Modern Method	5.0	18.4	31.2	41.5	43.4	47.3	47.5	52.1	54.1
Pill	2.7	5.1	13.9	20.8	23.0	26.2	28.5	27.2	27.0
IUD	0.5	1.4	1.8	1.8	1.2	0.6	0.9	0.7	0.6
Injectables	U	0.5	2.6	6.2	7.2	9.7	7.0	11.2	12.4
Implants	U	U	U	0.1	0.5	0.8	0.7	1.1	1.7
Vaginal Methods	0.0	0.2	U	U	U	U	U	U	U
Condom	0.7	1.8	2.5	3.9	4.3	4.2	4.5	5.5	6.4
Female Sterilization	0.6	7.9	9.1	7.6	6.7	5.2	5.0	5.0	4.6
Male Sterilization	0.5	1.5	1.2	1.1	0.5	0.6	0.7	1.2	1.2
Any Traditional Method	2.7	6.9	8.7	7.7	10.3	10.8	8.3	9.2	8.4
Periodic Abstinence	0.9	3.8	4.7	5.0	5.4	6.5	4.9	6.9	6.2
Withdrawal	0.5	0.9	2.0	1.9	4.0	3.6	2.9	1.9	1.9
Other Traditional Methods	1.3	2.2	2.0	0.8	0.9	0.6	0.6	0.4	0.3
Number of Women	U	7822	9745	8450	9720	10582	10192	16635	16858

N.B: U = unknown.

When it came to traditional family planning, periodic abstinence was a significant choice. Although only 0.9 percent of respondents used this method in 1975, 6.5 percent used it in 2004, 6.9 percent used it in 2011, and 6.2 percent used it in 2014.

In Bangladesh, the use of any long-acting or permanent contraception methods increased by one percentage point between 2007 and 2011 and remained at 8 percent between 2011 and 2014. Male sterilization and implant usage increased between 2007 and 2014, although the current use levels were low at 1 percent and 2 percent, respectively.

Along with an examination of the trend in the use of family planning via modern and traditional methods, a linear trend was also fitted to examine the CPR's incremental rate over a span of more than three decades. The regression coefficient's value indicates that there was an overall increase in CPR during the stated period.

**Figure 1:**
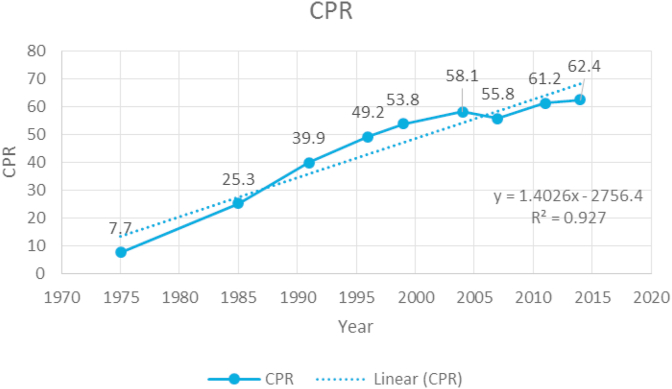
Linear trend line for the use of contraceptives during the period 1975–2014 in Bangladesh.

The observed and trend lines are presented in Figure 1 to assess the CPR pattern at a glance in the period 1975 to 2014. The trend line shows that there were positive increments during this period of, on average, 1.402 per every five years. The CPR trends for women with only daughters or only sons were computed as well and will be discussed in the next section.

## 4 Current contraceptive use

**Figure 2:**
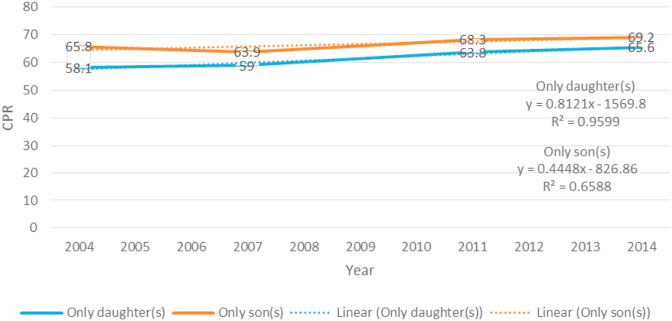
Trends in the use of contraceptives for respondents who had only daughters or sons from 2004 to 2014.

The observed percentages for the use of contraceptives are shown in Figure 2, which illustrates the trends in the distribution of contraceptive methods across the survey years among sexually active women in Bangladesh. It can be observed that overall contraceptive use among the respondents who had only daughters increased from 2004 to 2014. Their use rates were 58.1 and 65.6 percent in the years 2004 and 2014, respectively. A similar result can be seen for those respondents who had only sons; interestingly, there was a slight decrease in their CPR in 2007.

The linear trend was also fitted to understand the CPR's incremental rate over the course of four demographic surveys. The regression coefficient's value indicates that there was an overall increase in CPR during the stated period for those who had only daughters or sons.

Figure 2 shows that the contraceptive rate of those respondents who had only daughters was 0.812 over the period. In contrast, the rate was 0.448 for those who had only sons. These two increments hint that the respondents who had only sons took contraceptives more readily than the group who had only daughters. The data comparison indicates that respondents in Bangladesh prefer son(s) over daughters.

## 5 Results

In this section, contraceptive use and background characteristics are examined to identify the possible reasons for the preference for sons, and multivariate analysis is to show the odds of using a contraceptive method from one category as opposed to one from another category.

### 5.1 Percentage of respondents using contraceptive methods

The four BDHS datasets indicate that respondents who had only sons and higher levels of education practiced contraception at a higher rate than those who had only daughters with same level of education (Table 2). The highest contraception rate was found among more highly educated women in the 2014 BDHS, which were 70.3 and 69.3 percent, respectively, for those who had only sons and had only daughters. If a woman had a job, she was more likely to use contraceptive methods than her non-working counterparts. In 2011, 69.3 percent of working women who had only daughters used birth control, while the rate was 4.3 percentage points higher for those working women who had only sons. The patterns seen for women's educational backgrounds were also found for their husband's educational backgrounds in Bangladesh.

**Table 2:** Percentage distribution of the respondents using contraceptive methods by background characteristics.

Background Characteristic	Respondents who had only daughters				Respondents who had only sons			
	2004 BDHS	2007BDHS	2011 BDHS	2014 BDHS	2004 BDHS	2007 BDHS	2011 BDHS	2014 BDHS
**Respondent's Education**								
Illiterate	51.6	51.2	58.2	55.2	61.7	58.0	66.5	66.4
Primary	59.1	58.2	66.0	63.9	64.1	62.0	66.4	68.5
Secondary and more	61.9	62.6	64.7	69.3	70.1	67.8	70.0	70.3
**Respondent Currently Working**								
No	57.1	54.7	62.9	64.9	64.1	61.9	67.6	67.7
Yes	62.2	69.2	69.3	67.4	72.4	68.9	73.6	72.4
**Husband's Education**								
Illiterate	55.6	54.1	64.8	59.1	63.8	59.9	67.8	68.6
Primary	54.0	57.5	62.4	68.5	64.0	64.4	66.5	70.2
Secondary and more	62.9	62.9	64.0	66.8	68.5	66.2	69.7	68.9
**Place of Residence**								
Urban	67.0	66.3	68.4	70.1	73.2	69.7	71.4	73.5
Rural	55.1	56.6	61.9	63.5	63.4	62.1	67.0	67.3
**Religion**								
Muslim	57.7	58.7	63.4	64.7	64.4	63.1	67.5	68.0
Non-Muslim	62.3	61.9	66.8	74.4	75.4	71.4	74.4	78.0
**Access to Mass Media**								
No Access	48.4	50.6	62.0	61.8	57.5	56.6	67.7	66.7
Have Access	61.2	62.5	64.5	67.4	68.4	67.1	68.6	70.3
**Socioeconomic Conditions**								
Poor	54.3	56.2	64.5	64.3	61.0	63.0	68.6	69.8
Middle	56.2	62.1	62.7	68.0	63.5	60.3	69.4	69.6
Rich	62.1	59.8	63.7	65.6	71.1	66.1	67.7	68.6
**Number of Living only Daughter(s)**								
One Daughter	57.1	54.6	61.6	64.9	Not Applicable			
Two Daughters	60.3	64.9	67.1	70.3				
Three Daughters	60.8	67.3	64.5	63.2				
Four or More	52.1	52.7	66.8	55.1				
**Number of Living only Son(s)**								
One Son	Not Applicable				62.2	58.9	63.8	64.1
Two Sons					67.8	71.2	75.5	76.8
Three Sons					73.5	70.1	69.3	68.7
Four or More					69.5	56.5	61.3	75.0
**Overall**	58.1	59.0	63.8	65.6	65.8	63.9	68.3	69.2

Place of residence and religion are important determinants of the use of contraception. The highest CPRs of urban respondents were found in 2014, which were 70.1 and 73.5 percent for those had only daughters and had only sons, respectively. On the other hand, in the same year, among non-Muslim respondents, 74.4 and 78.0 percent used birth control methods when they had only daughters and had only sons, respectively.

#### 5.1.1 Mass media is a significant force in modern culture, particularly in developing countries

When women had access to mass media, the gap in the CPRs of the respondents who had only daughters and had only sons was about 3 percentage points in 2014 (67.4% vs. 70.3%), whereas the gap was 7.2 percentage points in 2004 (61.2% vs. 68.4%). Moreover, in terms of socioeconomic status, there is a 9 percentage point gap in contraceptive use between upper-class respondents in 2004 having only sons and only daughters, whereas that gap had reduced to 3 percentage points by 2014.

On the other hand, it should be pointed out that in 2014, only 55.1 percent of women who had four or more daughters only had used contraceptive methods, whereas 75 percent of women who had four or more sons only used contraceptives. The scenario is almost the same for the same number of offspring in the remaining three demographical surveys.

### 5.2 Multivariate analysis

Logistic regression analysis was used to examine the effects of the preference for sons on current contraceptive use via various independent categorical variables. The multivariate binary logistic regression analysis for the CPR among those with only daughters (group 1) and only sons (group 2) is shown in Table 3. The figures shown are exponentiated coefficients and thus indicate the relative odds of the use of contraceptives. The four survey datasets of the respondents who had only daughters indicate that those respondents with at least a secondary education had 1.35–1.90 times higher odds of using contraceptives than those had no education, whereas job holders had 1.21–1.88 times higher odds of practicing birth control than those had no job. Interestingly, similar results were found for the respondents who had only sons. On the other hand, the relative odds of practicing contraception was higher for the respondents whose husbands had at least a primary education than they were for those respondents whose husbands had no education (O.R = 1.27 and 1.07 for group 1 for 2014, and O.R = 1.21 and 1.10 for group 2 in 2007). Rural respondents were less likely to practice contraception than their urban counterparts (O.R = 0.67–0.71 for group 1 and 0.67–0.76 for group 2), whereas non-Muslim respondents were more likely to use contraceptive methods than their Muslim counterparts in all four survey years. In addition, access to mass media is a good indicator of the use of contraceptives. In the 2004 and 2007 survey years, respondents from group 1 who had access to any media type had 1.46 times and 1.45 times higher odds, respectively, of practicing contraception than those who had no access to mass media. On the other hand, respondents from group 2 who had access to mass media had higher odds of using contraception than those had no access to mass media (O.R = 1.28–1.50).

**Table 3:** Logistic regression analysis showing the odds ratios for respondents currently using contraception.

Background Characteristic	Group 1				Group 2			
	Respondents who had only daughters				Respondents who had only sons			
	2004 BDHS	2007 BDHS	2011 BDHS	2014 BDHS	2004 BDHS	2007 BDHS	2011 BDHS	2014 BDHS
Respondent's Education								
Illiterate	1.00	1.00	1.00	1.00	1.00	1.00	1.00	1.00
Primary	1.33∗∗	1.39∗∗	1.53∗∗∗	1.41∗∗	1.15	1.26∗	1.08	1.20
Secondary and more	1.35∗	1.73∗∗∗	1.60∗∗∗	1.90∗∗∗	1.46∗∗	1.76∗∗∗	1.39∗∗∗	1.57∗∗∗
Respondent Currently Working								
No	1.00	1.00	1.00	1.00	1.00	1.00	1.00	1.00
Yes	1.24∗	1.88∗∗∗	1.26∗∗	1.21∗∗	1.50∗∗∗	1.40∗∗∗	1.30∗∗	1.23∗∗∗
Husband's Education								
Illiterate	1.00	1.00	1.00	1.00	1.00	1.00	1.00	1.00
Primary	0.83	0.99	0.82∗	1.27∗∗	0.88	1.21∗	0.94	1.04∗
Secondary and more	1.06	1.22∗	0.87	1.07∗	0.86	1.10∗	1.05	0.91
Place of Residence								
Urban	1.00	1.00	1.00	1.00	1.00	1.00	1.00	1.00
Rural	0.68∗∗∗	0.67∗∗∗	0.71∗∗∗	0.70∗∗∗	0.74∗∗	0.76∗∗	0.74∗∗∗	0.67∗∗∗
Religion								
Muslim	1.00	1.00	1.00	1.00	1.00	1.00	1.00	1.00
Non-Muslim	1.17	1.11	1.17	1.56∗∗∗	1.61∗∗∗	1.52∗∗	1.36∗∗	1.57∗∗∗
Access to Mass Media								
No Access	1.00	1.00	1.00	1.00	1.00	1.00	1.00	1.00
Have Access	1.46∗∗∗	1.45∗∗∗	1.11	1.15	1.37∗∗	1.50∗∗∗	1.02	1.28∗∗∗
Socioeconomic Conditions								
Poor	1.00	1.00	1.00	1.00	1.00	1.00	1.00	1.00
Middle	0.92	1.07	0.85	0.93	1.04	0.78∗	0.94	0.89
Rich	0.97	0.71∗∗	0.75∗∗	0.69∗∗∗	1.23∗	0.75∗∗	0.70∗∗∗	0.68∗∗∗
Number of Living only Daughter(s)								
One Daughter	1.00	1.00	1.00	1.00	Not Applicable			
Two Daughters	1.22∗	1.61∗∗∗	1.32∗∗∗	1.36∗∗∗				
Three Daughters	1.30	1.97∗∗∗	1.23	1.03				
Four or More	0.96	1.16	1.49∗∗	0.89				

Number of Living only Son(s)								
One Son	Not Applicable				1.00	1.00	1.00	1.00
Two Sons					1.31∗∗	1.84∗∗∗	1.83∗∗∗	1.96∗∗∗
Three Sons					1.96∗∗∗	2.05∗∗∗	1.46∗∗∗	1.41∗∗
Four or More					1.67∗∗	1.30	1.08	2.22∗∗∗

Note: RC (1.00) = Reference Category, ∗p < 0.10, ∗∗p < 0.05; ∗∗∗p < 0.01.

There is some evidence that standard of living is associated with the use of contraceptives. The study shows that upper-class respondents had lower odds of using the birth control than the poor respondents in Bangladesh. Lastly, respondents who had only two living daughters had higher odds of using a contraceptive method than those who had only one daughter in all survey years (O.R = 1.22–1.61), whereas the odds of practicing contraception were 1.31–1.96 for the respondents who had only two sons compared to those who had only one son.

## 6 Discussion

This study's unique feature is the examination of the preference for sons in Bangladesh via an investigation into respondents who had only daughters or had only sons. The study also investigates the preference for sons from the perspective of demographical and social-economic variables correlated with the use of contraception. In general, women with more male children than female children were more likely to practice contraceptive methods than women with more female children than male children.

The educational attainment of a woman influences her decisions regarding her reproductive life. Based on the four survey datasets, the study found that the use of contraceptives was greater among educated women. Moreover, the study identified that respondents who had only sons practiced, on average, higher rates of contraception than those who had only daughters and this trend of contraceptive use was observed during the study year 2004–2014. This low CPR of women who had only daughters indicates that the preference for sons still prevails. Furthermore, the preference is severe among illiterate families. In addition to the woman's education, that of her husband is an important determinant in family size preferences, specifically the preference for sons. Examining gender preference via CPR follows Barber and Axinn (2004), who discovered that husbands' and wives' educations affected Nepal's gender preferences. Generally, higher levels of female and male education depress the preference for sons (Pande and Astone, 2007; Mason, 1997). It is widely accepted that more is to be spent on sons' educations than on daughters' educations in developing countries because a son's education perceived of as an investment or product that will yield future profit in the form of financial support in old age, while an investment in a daughter's education is considered to be an expenditure and a form of consumption. This philosophy leads to negative results for females, especially in the middle of or at the end of delivery when the sex of unborn baby is identified. Gipson and Hindin (2008), using cross-sectional and longitudinal survey data from Bangladesh, found that the likelihood of abortion depends on the composition of boys and girls in the family. In many Asian countries, sex-selective abortion has been reported (Gupta et al., 2009; Hesketh et al., 2011). Women's autonomy is an important factor in reduction of sex-selective abortions correspond to greatly increases the use of contraception. Thus, females' participation in the labor force is relevant to their autonomy (Bhattacharya, 2006; Rahman and Rao, 2004; Rahman et al., 2014). This study confirms this link in that it found that working women practiced a higher rate of birth control than non-working women. Interestingly, the preference for sons was still prevalent among women in the work force who had no sons.

This article also examined the preference for sons in rural areas of Bangladesh, which is a burning issue. In Bangladesh, a lower-middle-income country, the huge urban-rural disparity is significant in economically, politically, and in term of opportunities. The preference for sons is very problematic in the rural areas of Bangladesh. This study found that women who had only daughters and lived in remote areas used contraception at lower rates than those with only sons and also lived in the same place. In addition, Muslim women who had only daughters used birth control at a lower rate than those with no daughters. The low CPR of Muslim women confirms the presence of the preference for sons. These findings are similar to the findings of other studies that took place in Bangladesh (Hoq, 2019; Visaria, 2015; Alam et al., 2018).

Media exposure also has a potential effect on contraceptive use and influences the attitudes of women towards the preference for sons. In a study on India, a similar country of Bangladesh, Jensen and Oster (2009) reported that the role of mass media increased women's autonomy and decreased cases of domestic violence against women, gender bias, and the preference for sons. This study found that women who had access to any type of mass media used family planning at higher rates than those who had no access to media and that the rate was higher among those women who had only sons compared to those who had only daughters. More specifically, there was preference for sons among the women who had no sons, which supports Jensen and Oster (2009) findings.

Various empirical studies have examined the relationship between the preference for sons and socioeconomic status. These studies identified that increasing wealth significantly reduced the preference for sons (Murphy et al., 2011; Gaudin, 2011; Talukder et al., 2014). Generally, the wealthiest families had lower odds of gender preferences (Fuse, 2008). Surprisingly, this study found the lowest odds of contraceptive use among the wealthiest couples who had no sons when poor couples were used as the reference category, which is opposite the findings of Fuse (2008) for several North African and Asian countries. The possible reason for this unexpected finding is preferences related to the gender composition of families. Indeed, the study found that reproductive decisions are influenced by the number of sons and daughters in the family. More explicitly, women with at least four daughters and no sons had a relatively lower CPR than those who had fewer than four daughters and no sons, while the rate was higher for those with at least four sons and no daughters. These scenarios point out that parents who already have some daughters would also prefer to have some sons, thus influencing the relationship between the number of sons and the use of contraceptives (Ritchie and Roser, 2019). Dahal et al. (2008) found that men in Nepal who had at least two living sons were more likely to have used permanent methods of contraception when compared to men who had only daughters. The woman's number of surviving children has a substantial effect on her present contraception utilization in comparison, which may indicate that a woman utilizes contraceptives when she feels that she has enough children, especially sons.

## 7 Conclusion and policy implications

The prevalence of the preference for sons is important for achieving an understanding of the underlying determinants of the low use of contraceptives due to its possible influence on family size in Bangladesh. Gender preferences and the composition of boys and girls in their families were the major reasons why women intended to bear more children after producing the mean perceived ideal number (2.22) of living children (NIPORT, 2016). Several factors, including education, employment, place of residence, religion, mass media, socioeconomic status, and the number of living daughters and sons were significant factors in the use of contraceptives among Bangladeshi women.

The findings have important policy implications for the family planning program in Bangladesh. Electronic and print media campaigns targeting couples should be launched to discourage the preference for sons. Moreover, sustained efforts be made to raise awareness about gender equality and proper use of contraceptives. Increasing the literacy rate, creating job opportunities, increasing women's autonomy, creating easy access to electronic and print media, improving the socioeconomic status of women, and narrowing the gender gap in Bangladesh would all help. Thus, an effective public awareness campaign in which sons and daughters are treated equally would be beneficial in securing a balance between male and female offspring.

## Declarations

### Author contribution statement

Mohammad Nazmul Hoq: Conceived and designed the experiments; Performed the experiments; Analyzed and interpreted the data; Contributed reagents, materials, analysis tools or data; Wrote the paper.

### Funding statement

This research did not receive any specific grant from funding agencies in the public, commercial, or not-for-profit sectors.

### Competing interest statement

The authors declare no conflict of interest.

### Additional information

No additional information is available for this paper.
